# Repeated disinfectant use in broiler houses and pig nursery units does not affect disinfectant and antibiotic susceptibility in *Escherichia coli* field isolates

**DOI:** 10.1186/s12917-020-02342-2

**Published:** 2020-05-18

**Authors:** H. Maertens, E. Van Coillie, S. Millet, S. Van Weyenberg, N. Sleeckx, E. Meyer, J. Zoons, J. Dewulf, K. De Reu

**Affiliations:** 1 Flanders Research Institute for Agriculture, Fisheries and Food (ILVO), Technology and Food Science Unit, Brusselsesteenweg 370, 9090 Melle, Belgium; 2 Experimental Poultry Center (EPC), Poiel 77, 2440 Geel, Belgium; 3 Veterinary Biochemistry Unit, Department of Pharmacology, Toxicology and Biochemistry, Faculty of Veterinary Medicine, Ghent University, Salisburylaan 133, 9820 Merelbeke, Belgium; 4 Veterinary Epidemiology Unit, Department of Reproduction, Obstetrics and Herd Health, Faculty of Veterinary Medicine, Ghent University, Salisburylaan 133, 9820 Merelbeke, Belgium

**Keywords:** Disinfectants, Antibiotic resistance, Disinfectant susceptibility, *Escherichia coli*, Pigs, Poultry

## Abstract

**Background:**

Disinfectants are frequently used in animal production to reduce or eliminate the load of infectious agents and parasites in buildings and equipment associated with the housing or transportation of animals. There are growing concerns that the use of disinfectants would select for resistance to antibiotics and disinfectants. The aim of this study was to determine the effect of repeated use of different disinfectants on the disinfectant and antibiotic susceptibility under practical conditions in a broiler and pig pilot farm. Therefore, the susceptibility of *Escherichia coli* (*E. coli*) to 14 antibiotics and 4 disinfectants was monitored over a one-year period.

**Results:**

High (20–50%) to very high (> 50%) resistance levels for ampicillin, sulfamethoxazole, trimethoprim and tetracycline were observed in both animal production types. Disinfectant susceptibility did not change over time and did not depend on the used disinfection product. Compared to in-use concentrations of formaldehyde, benzalkoniumchloride and a peracetic acid - hydrogen peroxide formulation, all *E. coli* strains remained susceptible indicating that the use of disinfectants did not select for disinfectant resistance. Moreover, no association could be found between the use of disinfectants and antibiotic resistance.

**Conclusions:**

These findings suggest that repeated use of disinfectants in agricultural environments does not select for antibiotic resistance nor does it reduce disinfectant susceptibility.

## Background

Biocides are used in animal production to disinfect buildings and equipment associated with the housing or transportation of animals. Their appropriate use preceded by an adequate cleaning is one of the key elements of a good on-farm hygiene management. Cleaning and disinfection (C&D) are of importance to reduce the introduction and spread of infectious agents. Cleaning refers to the removal of organic debris as its presence can decrease the antimicrobial activity of the disinfectant. Disinfection reduces or eliminates the load of bacteria and viruses [1], yet bacteria may still be present after C&D of animal houses [2]. This has led to the hypothesis that bacteria can become resistant to the used disinfectants. In recent years, studies have therefore focused on the disinfectant susceptibility of field bacterial isolates, but the methodologies used to evaluate the susceptibility data lead to heterogeneous results. Evaluation of the frequency distribution of the minimal inhibitory concentration (MIC) and/or minimal bactericidal concentration (MBC) is a first method to investigate resistance. When a homogenous distribution is shown, there is no indication for a reduced susceptibility [3, 4]. Secondly, the lethality of in-use disinfectant concentrations is evaluated via MBC determinations. Both susceptible and less susceptible field isolates to in-use concentrations have been reported [5]. Thirdly, MIC_90_ values calculated as the disinfectant concentration that inhibits 90% of the field isolates are compared with MIC values of a control strain. Reports have shown both similar and reduced susceptibilities [6]. Lastly, concentration criteria are used to categorize isolates as either susceptible, low-level resistant, or resistant according to their MIC. For example, a low-level resistance prevalence to benzalkoniumchloride has been found [7].

Furthermore, contradictory results have been found regarding the possible association between the reduced susceptibility to disinfectants and antibiotic resistance in field bacteria [8–11]. Moreover, few studies were performed on the use of disinfectants and the relation to antimicrobial resistance under practical conditions [12].

In exposure experiments under laboratory conditions, it has been shown that stepwise repeated exposure of susceptible bacteria to subinhibitory concentrations of disinfectants may lead to decreased susceptibility to various antimicrobial agents [13–17]. However, many of these investigations do not relate such findings to practical conditions and the laboratory results are not necessarily relevant to agricultural environments. Moreover, few studies investigated changes in antimicrobial susceptibility over time in a practical setting [18]. Therefore, this longitudinal study was carried out to investigate the effect of repeated disinfectant use on antimicrobial susceptibility of *Escherichia coli* (*E. coli*), isolated after C&D in broiler houses and pig nursery units. A broiler is any chicken that is bred and raised specifically for meat production.

## Results

### Detection of *Escherichia coli*

In the broiler house, positive swabs were detected after disinfection with Virocid^®^, CID20^®^ and D50^®^ in 9.7, 8.6 and 20.1% of the locations, respectively. In the pig nursery unit, positive swabs were detected in 47.7, 22.9 and 36.8% of the locations, respectively.

### Disinfectant susceptibility

#### Disinfectant use and susceptibility

**Fig. 1:**
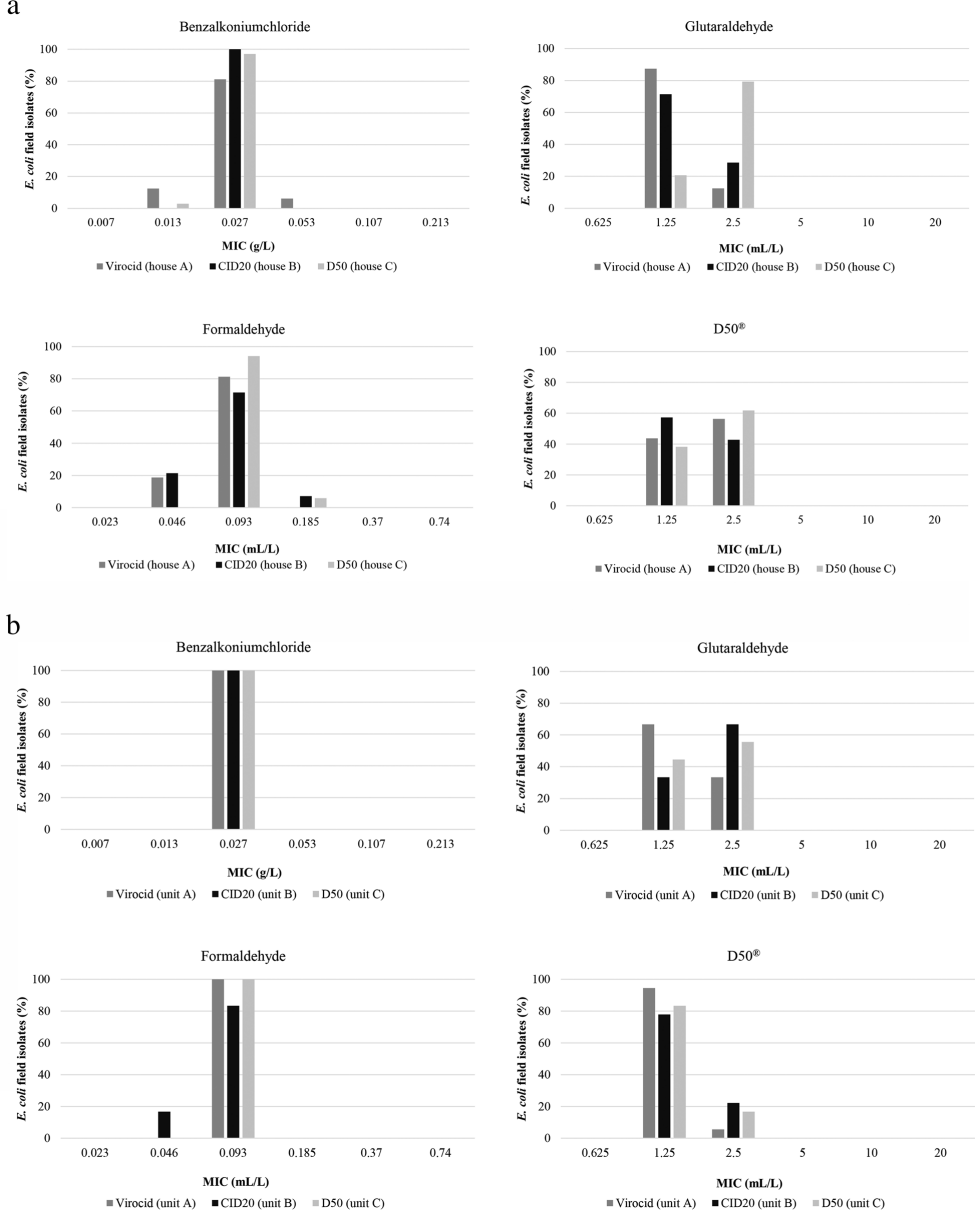
**a** Percentage of *Escherichia coli* field isolates with their respective minimum inhibitory concentrations (MICs) displayed on the horizontal axis for benzalkoniumchloride, glutaraldehyde, formaldehyde and D50^®^, isolated after the disinfection protocols with Virocid^®^ (*n* = 16), CID20^®^ (*n* = 14) or D50^®^ (*n* = 34) at the corresponding broiler houses A, B and C, respectively. **b** Percentage of *Escherichia coli* field isolates with their respective minimum inhibitory concentrations (MICs) displayed on the horizontal axis for benzalkoniumchloride, glutaraldehyde, formaldehyde and D50^®^, isolated after the disinfection (t1, t3 and t5) with Virocid^®^ (*n* = 18), CID20^®^ (*n* = 18) or D50^®^ (*n* = 18) at the corresponding pig nursery units A, B and C, respectively

The *E. coli* isolates (*n* = 67 from the broiler houses, *n* = 72 from the pig nursery units), obtained after disinfection were tested for their susceptibility to benzalkoniumchloride, glutaraldehyde, formaldehyde and D50^®^. Disinfectant susceptibility results were homogeneously (normally) distributed within a very small concentration range for all disinfectants tested (Fig. 1a and b). No remarkable differences in MICs were found between the *E. coli* isolates, either with the various disinfectants or between both animal production types.

#### Disinfectant susceptibility evolution

**Fig. 2:**
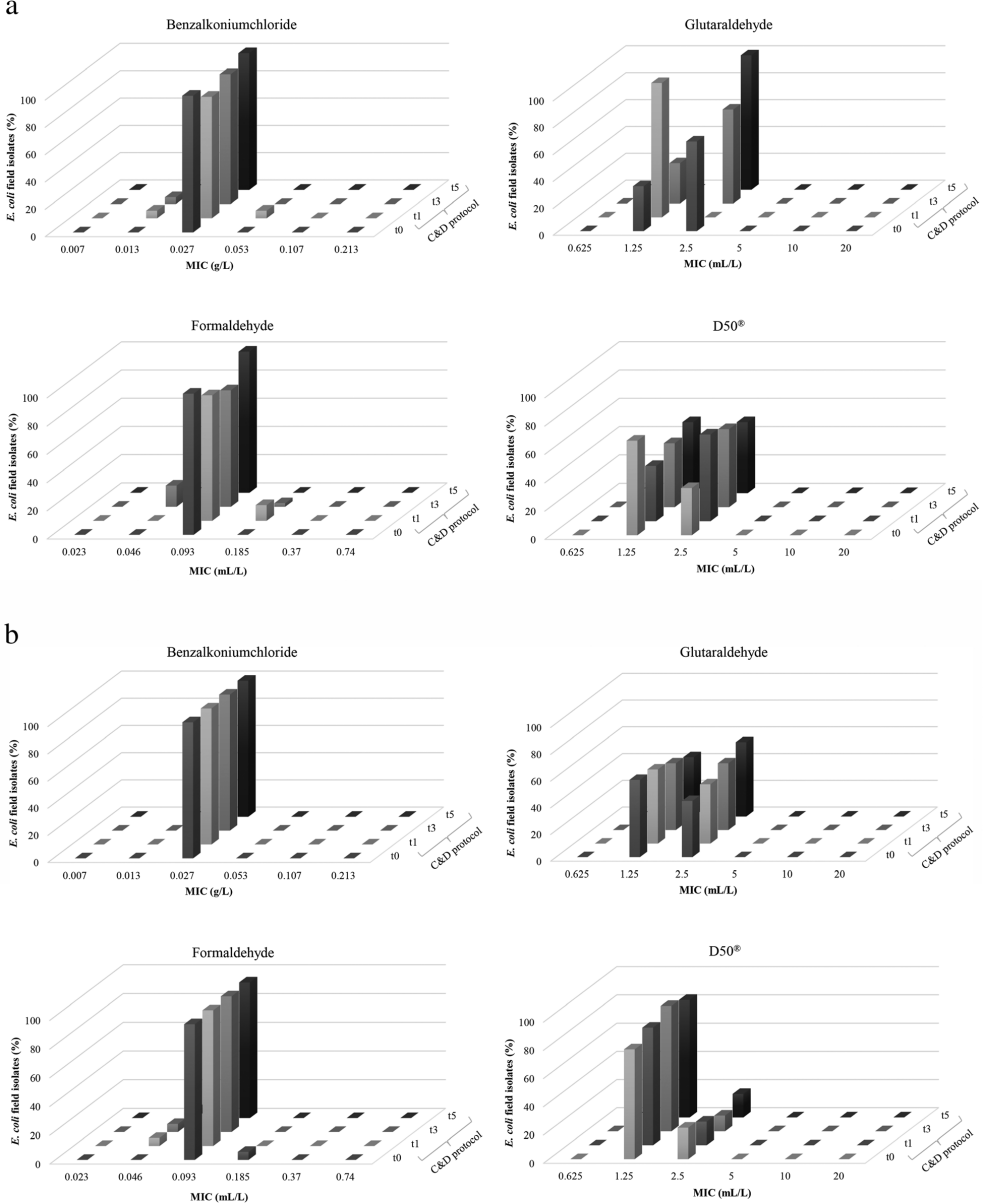
**a** Evolution in minimum inhibitory concentrations (MICs) of *Escherichia coli* field isolates for benzalkoniumchloride, glutaraldehyde, formaldehyde and D50^®^, expressed as percentages. Samples were taken after cleaning and disinfection (C&D) over a period of 6 production cycles: t0 (zero measurement, *n* = 3), t1 (after production cycle 1, *n* = 18), t3 (after production cycle 3, *n* = 40) and t5 (after production cycle 5, *n* = 6) at the pilot broiler farm. Monitoring of C&D took place from production cycle 1. **b** Evolution in minimum inhibitory concentrations (MICs) of *Escherichia coli* field isolates for benzalkoniumchloride, glutaraldehyde, formaldehyde and D50^®^, expressed as percentages. Samples were taken after cleaning and disinfection (C&D) over a period of 6 production cycles: t0 (zero measurement, *n* = 18), t1 (after production cycle 1, *n* = 18), t3 (after production cycle 3, *n* = 18) and t5 (after production cycle 5, *n* = 18) at the pilot pig farm. Monitoring of C&D took place from production cycle 1 onwards

No change in susceptibility to benzalkoniumchloride, glutaraldehyde, formaldehyde and D50^®^ could be observed over time (Fig. 2a and b). Furthermore, as all *E. coli* isolates showed a similar susceptibility to the active components benzalkoniumchloride, glutaraldehyde, formaldehyde and D50^®^, no indications for disinfectant resistance were found.

### Antibiotic resistance

**Fig. 3:**
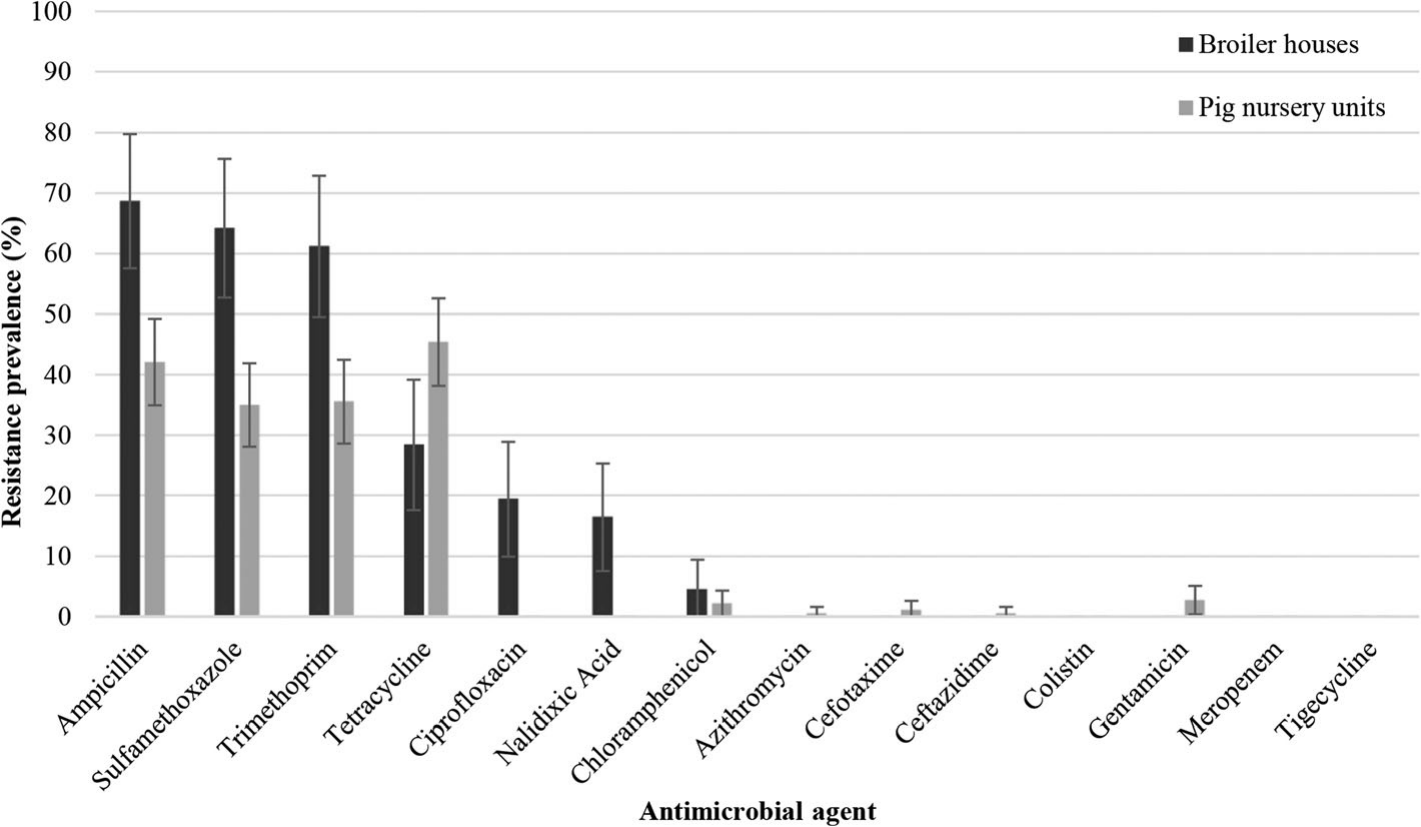
Prevalence of antibiotic resistance in 67 and 183 *Escherichia coli* isolated at the 4 sampling moments (t0: zero measurement, t1: after production cycle 1, t3: after production cycle 3 and t5: after production cycle 5) after cleaning and disinfection over 6 production cycles in 3 broiler houses and 3 pig nursery units, respectively (expressed as percentage). Error bars represent the standard errors

The 67 *E. coli* isolates from broiler houses and the 183 isolates from pig nursery units were exposed to a panel of 14 antibiotics to evaluate antibiotic susceptibility (Fig. 3). Occurrence of antibiotic resistance in the broiler houses was very high for ampicillin (69%), sulfamethoxazole (64%) and trimethoprim (61%). A high and moderate antibiotic resistance was found for tetracycline (28%), ciprofloxacin (19%) and nalidixic acid (16%). Resistance toward chloramphenicol was low (4%). No resistance was found to the other tested antibiotics.

For *E. coli* isolates from pig nursery units, high levels of antibiotic resistance to tetracycline (45%), ampicillin (42%), trimethoprim (36%) and sulfamethoxazole (35%) were found. Antibiotic resistance to gentamicin and chloramphenicol was low. Very low to no resistance was found for the other tested antibiotics.

#### Disinfectant use and antibiotic resistance

**Fig. 4:**
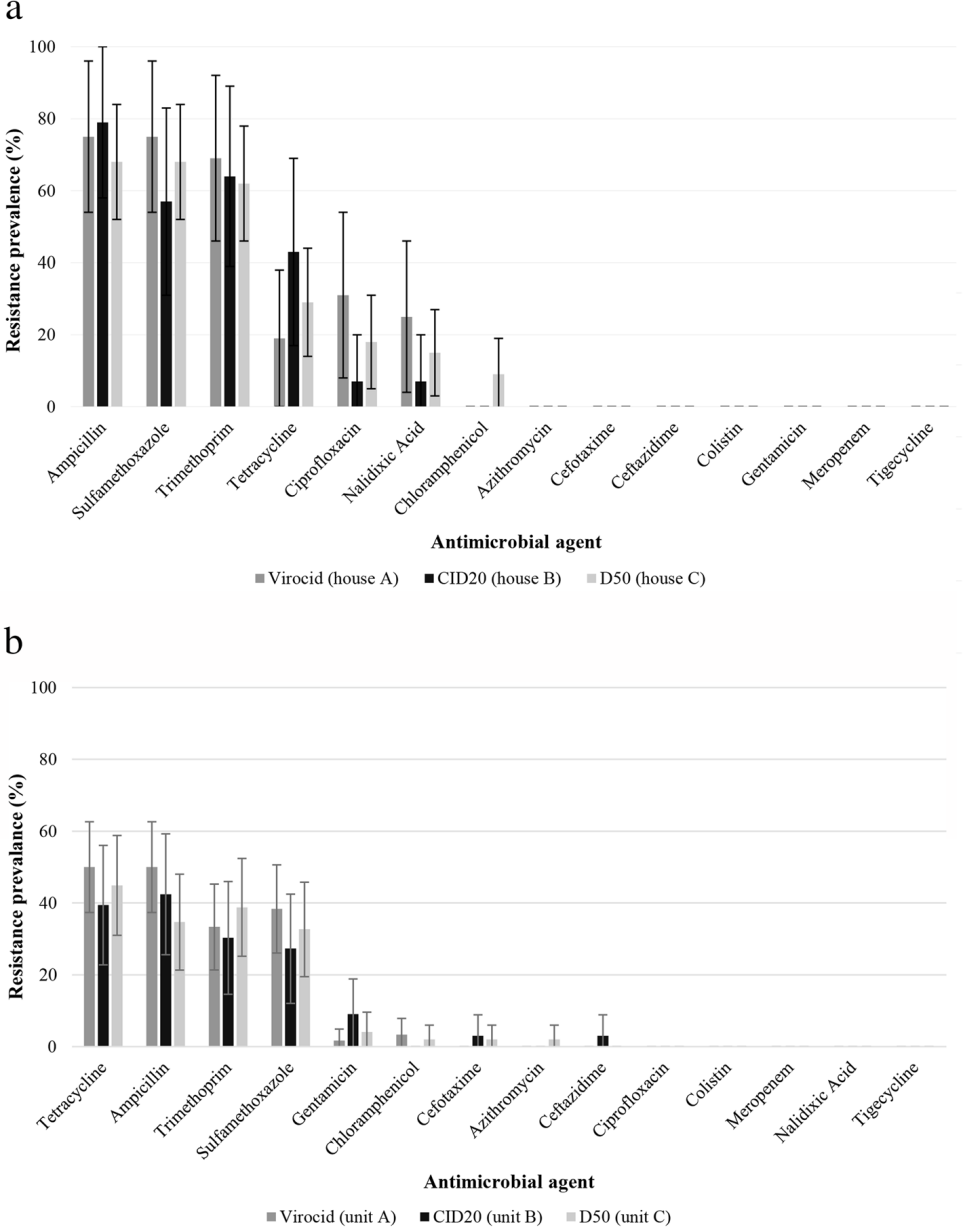
**a** Prevalence of antibiotic resistance in *Escherichia coli* isolated after the disinfection protocols with Virocid^®^ (*n* = 16), CID20^®^ (*n* = 14) or D50^®^ (*n* = 34) at the corresponding broiler houses, sampled 3 times (t1: after production cycle 1, t3: after production cycle 3 and t5: after production cycle 5) (expressed as percentage). Error bars represent the standard errors. **b** Prevalence of antibiotic resistance in *Escherichia coli* isolated after the disinfection protocols with Virocid^®^ (*n* = 60), CID20^®^ (*n* = 33) or D50^®^ (*n* = 50) at the corresponding pig nursery units, sampled 3 times (expressed as percentage). Error bars represent the standard errors

The antibiotic resistance prevalence of *E. coli*, isolated after each disinfection protocol in the corresponding broiler houses and pig nursery units A, B and C is presented in Fig. 4a and b. Logistic regression analysis showed no significant association between the used disinfectants and the antibiotic resistance of the isolates (Supplementary Table 1).

#### Antibiotic susceptibility evolution

An increasing trend in antibiotic resistance to ampicillin, sulfamethoxazole and trimethoprim was found between sampling t1 and t3 at the broiler pilot farm (Supplementary Fig. 1a). This increase corresponds to the antibiotic treatment of all broiler chickens with penicillins and sulfamides-trimethoprim within this time period (Supplementary Table 2). Furthermore, a clear increase in resistance was also noticed between t3 and t5 for tetracycline, ciprofloxacin and nalidixic acid at the broiler farm. In this particular period of time the hatchery origin of the one-day old broiler chickens changed.

For the *E. coli* field isolates obtained at the pig pilot farm, no trend in antibiotic resistance was observed during the study (Supplementary Fig. 1b).

## Discussion

To our knowledge, this is the first longitudinal field study on the effect of repeated use of disinfectants on the antimicrobial susceptibility of *E. coli* field isolates in agricultural environments.

### Disinfectant use and susceptibility

In the current study no evidence was found of reduced susceptibility to the tested active components following disinfection with three different disinfectants at either the broiler houses or the pig nursery units. The MIC values for the disinfectant components were homogenously distributed within a very small concentration range without a bimodal distribution. Furthermore, MIC values of the *E. coli* field isolates for the disinfectant components were similar to those recently reported by our research group [19]. The obtained MIC values of two active components i.e. benzalkoniumchloride and formaldehyde were compared to the in-use concentrations in two evaluated veterinary disinfection products i.e. Virocid^®^ and CID20^®^. These MIC values were lower than the recommended concentrations of benzalkoniumchloride and formaldehyde in veterinary disinfection products applied by foaming. This difference indicates that the prescribed commercial product concentrations are sufficiently high to reduce the bacterial load with at least 5 log CFU. In contrast, when comparing the obtained MIC value of the third active component i.e. glutaraldehyde to the in-use concentration of this active component in two of the evaluated veterinary disinfection products i.e. Virocid^®^ and CID20^®^, the former MIC value was much higher than the recommended in-use concentration in veterinary disinfection products applied by foaming. However, in practice, aldehyde-based disinfectants are formulated in combination with quaternary ammonium compounds (QACs) such as in Virocid^®^ and CID20^®^ to achieve a synergistic effect [20, 21]. Susceptibility results for the ready-to-use disinfectant D50^®^, being a peracetic acid and hydrogen peroxide formulation, showed MIC values equal to or below the recommended concentration.

### Disinfectant susceptibility evolution

Reported in vitro disinfectant susceptibility data for bacteria are diverging. Several in vitro studies have shown a reduced susceptibility to disinfectants after repeated exposure of bacteria to subinhibitory concentrations of QACs [15, 22] or even a commercial disinfectant [23]. However, other in vitro studies state the opposite: in the studies of Karatzas et al. (2007) and Webber et al. (2015), with commercially available disinfectants instead of active components, none of the adapted strains demonstrated an increased tolerance after exposure to the commercial disinfectants [13, 16]. In the current in vivo study, repeated use of the same disinfectant during 5 production cycles did not show changes in disinfectant susceptibility over the (short) monitored time period to either the single active components benzalkoniumchloride, formaldehyde, glutaraldehyde or to the combined formulation D50^®^. Nevertheless, it should be borne in mind that resistance development requires time. The study was carried out on a short term and therefore some caution is warranted in interpreting the results.

It can be hypothesized that the reason for surviving strains after C&D is not resistance to the disinfectant, but could be (i) an inadequate C&D at critical locations in the animal production unit which are difficult to C&D, and/or (ii) residual organic matter, and/or (iii) other factors influencing the efficacy of disinfectants (e.g. disinfectant dilution by remaining rinsing water, environmental temperature). Corroborating this hypothesis, we previously reported that the disinfection of farm buildings and equipment does not lead to sterile surfaces and environments [24].

### Disinfectant use and antibiotic resistance

In the current in vivo study no evidence of reduced susceptibility to the tested antibiotics could be found following repeated disinfection with different disinfectants at the broiler houses and pig nursery units. This is again in contrast to several in vitro studies where a reduced susceptibility to antibiotics after repeated exposure of bacteria to subinhibitory concentrations of an active component [15, 22] or even of commercial disinfectants [13, 16, 23] was shown. The question is how to interpret this subset of data. They at least strongly suggest that, although disinfectant use may lead to an increased antibiotic resistance in vitro, it does not induce the development of such resistance in an agricultural environment.

### Antibiotic resistance evolution

Still, changes in antibiotic resistance were noticed even if logistic regression analysis showed that these high resistance levels were not associated with the use of disinfectants. The increasing antibiotic resistance level to ampicillin, sulfamethoxazole and trimethoprim between sampling t1 and t3 at the broiler pilot farm corresponds to the antibiotic treatment of all broiler chickens with penicillins and sulfamides-trimethoprim within this time period. Such association has already been described by our group and others [25, 26]. Furthermore, the most likely explanation for the high levels of antibiotic resistance to tetracycline, ciprofloxacin and nalidixic acid at the broiler farm at t5 is a change in hatchery origin of the one-day old broiler chickens, and the age of the parent breeding hens between t3 and t5. Unfortunately, no information was available on antibiotic treatments applied in the hatcheries. Last but not least, very high resistance levels of > 50% were found for ampicillin, sulfamethoxazole, trimethoprim, tetracycline, ciprofloxacin and nalidixic acid in the broiler isolates and high resistance levels of >20% to ampicillin, sulfamethoxazole, trimethoprim and tetracycline in the pig isolates. Compared to the Belgian report by CODA-CERVA (2017) on *E. coli* in 2016 [27], a lower resistance prevalence was found for both animal production types in our longitudinal study, except for trimethoprim in the broiler pilot farm. However, it should be noted that a limitation of the current study is that it only comprises results of one experimental broiler and one pig farm, which is likely not representative for the average Belgian broiler and pig farm.

## Conclusion

In conclusion, even if some in vitro studies previously reported a decreased antibiotic and/or disinfectant susceptibility after repeated exposure to disinfectants, we could not confirm this observation in our longitudinal field study. Indeed, the repeated use of disinfectants in recommended concentrations in vivo did not influence disinfectant susceptibility of *E. coli* isolated from broiler and pig units. Furthermore, the observed disinfectant susceptibility was also product-independent. Finally, no association was found between the use of disinfectants in both agricultural environments and the antibiotic resistance of the broiler and pig farm *E. coli* strains.

## Methods

### Management of C&D in the broiler houses and pig nursery units

The longitudinal study was carried out in three identical broiler houses at a pilot farm for broiler chickens (± 6000 broilers/house) of the Experimental Poultry Centre (EPC, Geel, Belgium) and in three identical pig nursery units (8 pens with 6 piglets per pen) at the experimental pig farm of the Flanders Research Institute for Agriculture, Fisheries and Food (ILVO) for 6 successive production cycles between May 2017 and May 2018. After the first production cycle, conventional C&D was carried out as follows: cleaning of the three broiler houses was applied by high pressure cleaning with a detergent (Kenosan^®^, CID LINES, Ieper, Belgium) and disinfection was applied by fogging with a quaternary ammonium compound, glutaraldehyde and formaldehyde based disinfectant (CID20^®^, CID LINES). In the pig nursery units high pressure cleaning was performed only with hot water (± 65 °C) followed by a disinfection with quaternary ammonium compounds and glutaraldehyde (MS Megades^®^, MS Schippers, Bladel, The Netherlands) via foaming. Subsequently, different C&D protocols were applied during 5 successive production cycles. Cleaning of all broiler houses and pig nursery units was carried out by high pressure cleaning with Kenosan^®^ and disinfection was applied by fogging at the broiler houses and by foaming at the pig nursery units. Selection of three commercially available disinfectants was based on frequently used combinations of active components in poultry houses and pig nursery units, described by Maertens et al. (2018, 2019) [19, 28]. Each disinfection product was applied during 5 successive production cycles in the same broiler house or pig nursery unit. The applied disinfection products consisted of (A) quaternary ammonium compounds and glutaraldehyde (QAC-GA; Virocid^®^, CID LINES), (B) quaternary ammonium compounds, glutaraldehyde and formaldehyde (QAC-GA-F; CID20^®^, CID LINES) and (C) peracetic acid and hydrogen peroxide (PA-H_2_O_2_; D50^®^, CID LINES). An overview of the used products, methods and product concentrations is given in Table 1.

**Table 1:** Cleaning and disinfection (C&D) protocols carried out at the start of the longitudinal study (conventional C&D) followed by C&D with 3 C&D protocols (A, B and C) carried out during 5 successive production cycles in each of the broiler houses or pig nursery units at the pilot farms

**Conventional C&D (sampling after production cycle 0)**			**Broiler house (1050 m**^3^)			**Pig nursery unit**		
			**A**	**B**	**C**	**A**	**B**	**C**
	**Cleaning**	Product	Kenosan^®^			No cleaning product		
		Concentration	1%					
		Method	foaming					
	**Disinfection**	Product	CID20^®^			MS Megades^®^		
		Concentration	3 l / 6 l water			1%		
		Method	fogging			foaming		
		Active components^*^	QAC-GA-F			QAC-GA		
**C&D protocol (sampling after production cycle 1, 3 and 5)**			**Broiler house**			**Pig nursery unit**		
			**A**	**B**	**C**	**A**	**B**	**C**
	**Cleaning**	Product	Kenosan®			Kenosan®		
		Concentration	1%			1.5%		
		Method	foaming			foaming		
	**Disinfection**	Product	Virocid^®^	CID20^®^	D50^®^	Virocid^®^	CID20^®^	D50^®^
		Concentration	2 l / 4 l water	4 l / 4 l water	4 l / 8 l water	0.25%	0.5%	0.5%
		Method	fogging	fogging	fogging	foaming	foaming	foaming
		Active components^*^	QAC-GA	QAC-GA-F	PA-H_2_O_2_	QAC-GA	QAC-GA-F	PA-H_2_O_2_

^*^QAC quaternary ammonium compound, *GA* glutaraldehyde, *F* formaldehyde, *PA* peracetic acid, *H*_*2*_*O*_*2*_ hydrogen peroxide

### Quantification of antibiotic use

The antibiotic use at the sampled pilot farms was recorded via prescriptions and order forms. For each treatment, the product name, the amount of administration and the age (days) and weight (kg) of treated animals were recorded. Quantification of drug use was done as described in Maertens et al. (2019) [19] by determining the treatment incidence (TI) defined as the number of treatment days per 100 days or the percentage of treatment days [29].

### Sampling and sampling processing

Sampling was performed ±24 h after disinfection at each broiler house or pig nursery unit at the following moments: t0 (zero measurement, after production cycle 0 = conventional C&D), t1 (after production cycle 1), t3 (after production cycle 3) and t5 (after production cycle 5) (Table 1). Sponge swabs were pre-moistened with 10 mL Dey Engley Neutralizing Broth (Sigma Aldrich, D3435, St-Louis, USA). Permission to collect samples was obtained from the EPC and the ILVO.

At each broiler house eight locations (floor, floor crack, drain hole, air inlet, drinking cups, pipes, wall and feed pan) were swabbed 8 times, except for the drain holes since only 2 drain holes were present in each broiler house, resulting in 58 samples. As three houses were included in the study, 174 samples were taken per sampling thus obtaining a total of 696 samples after 4 sampling moments.

For each pig nursery unit, each pen was sampled at six different locations (floor, concrete wall, synthetic wall, feeding trough, drinking nipples and pipes) resulting in 48 samples per pig nursery unit, hence 144 samples per sampling moment and a total of 576 swab samples during the entire longitudinal study. Whenever possible, a surface of 625 cm^2^ was swabbed. Since the surface of the drinking cups and nipples was smaller than 625 cm2, a total amount of five and two were swabbed at each broiler house and pig nursery unit, respectively. After sampling, swab samples were transported to the lab in a cool box with ice packs.

### Detection and isolation of *Escherichia coli*

On the same day, each swab sample was enriched with 10 mL of Buffered Peptone Water (BPW, Oxoid, CM0509, Basingstoke, Hampshire, England), homogenized by a Masticator (IUL instruments, S.A., Barcelona, Spain) and incubated for 24 h at 37 °C for the detection of *E. coli*. After incubation, 10 μL of the BPW fraction was plated on Rapid’*E. coli* 2 agar plates (Bio-Rad, 356–4024, Marnes-la-Coquette, France) and incubated at 44 °C for 24 h. From each positive Rapid’*E. coli* 2 plate one isolate was purified and stored at − 80 °C on brain heart infusion (BHI, Oxoid, CM1032) supplemented with 15% (v/v) glycerol. In total, 67 and 183 *E. coli* isolates were obtained during the longitudinal study at the broiler pilot farm and the experimental pig farm, respectively.

### Disinfectant susceptibility testing

#### Isolate and disinfectant selection

All 67 isolates from the broiler pilot farm were selected for disinfectant susceptibility testing. For the isolates from the experimental pig farm, a random selection was made of 6 isolates for each sampling and each sampled pig nursery unit, resulting in 72 pig isolates, representing ±40% of the total amount.

In our previous study [19] no difference was observed for the selected disinfectants between the MIC (minimal inhibitory concentration = minimal concentration that inhibits growth) and MBC (minimal bactericidal concentration = minimal concentration that results in ~ 5 log CFU reduction). Therefore, in the current study only the MICs were determined. Based on the used disinfectants at the pilot farms, active components present in the disinfectants were selected. These are: alkyldimethylbenzylammoniumchloride (BKC, > 95%, Sigma Aldrich, 12,060), formaldehyde (F, 35% v/v in H_2_O, Sigma Aldrich, 252,549), glutaraldehyde (GA, 50% w/v in H_2_O, Sigma Aldrich, 3802) and a chemically stable formulation of peroxyacetic acid (PA, 55 g/L) and hydrogen peroxide (H_2_O_2_, 220 g/L) (D50^®^, CID LINES, Ieper, Belgium) as hydrogen peroxide is not stable and rapidly degrades into water and oxygen and PA can also decompose to acetic acid and oxygen [30].

#### Inoculum preparation

Preparation of the inoculum was based on Maertens et al. (2019) [19].

#### Minimal inhibitory concentration (MIC)

Through a broth micro-dilution method based on the method described by Knapp et al. (2015) [31], the MICs of each active component (BKC, F and GA) or given formulation (D50^®^) were determined for the selected isolates. The MIC was defined as the lowest concentration of active components or formulation where no growth was visually observed. A 96-well microtiter plate with U-shaped wells (Novolab, A19652) was filled with 50 μL TSB containing twofold dilutions of the active component or formulation. Fifty microliters of the field isolates (1–5 × 10^8^ CFU bacterial /mL) was added to the TSB in the microtiter plate, resulting in a total volume of 100 μL. Final concentration ranges were as follows: 0.007–0.213 g/L BKC, 0.023–0.740 mL/L F, 0.625–20 mL/L GA and 0.625–20 mL/L D50^®^. As a positive control, 50 μL of each bacterial suspension was added to 50 μL TSB without disinfectant. To check for possible contamination, wells without bacterial suspension and disinfectant served as blank. After inoculation, plates were incubated for 24 h in a shaking incubator (100 rpm) at 37 °C. After incubation, the MICs were read. In every experiment the *E. coli* ATCC strains 10536 and 25922 were used as controls.

### Antibiotic susceptibility testing

Antibiotic susceptibility testing was performed on all *E. coli* broiler pilot farm isolates (*n* = 67) and experimental pig farm isolates (*n* = 183), using a microdilution method (Sensititre^®^) based on Maertens et al. (2019) [19].

### Data analysis

For both animal categories, antibiotic resistance data of the *E. coli* isolated after disinfection were each grouped for every sampling moment and disinfectant. A binary logistic regression model was fitted to the data with the antibiotic resistance profile at herd level (resistant/susceptible) as the dichotomous dependent variable and with the applied disinfectants, moment of sampling (t1, t3 and t5) and antibiotic use (TI100) as independent variables. *P*-values ≤0.05 were considered to be significant. All statistical analyses were performed using the Statistical Package for the Social Sciences (SPSS Statistics 25.0, IBM Corporation, Armonk, NY). Data of the conventional C&D (sampling at t0) were not included in the analysis.

## Supplementary information

**Additional file 1: Supplementary Table 1.** Statistical output of the logistic regression analysis.

**Additional file 2: Supplementary Fig. 1a and b.** Prevalence of antibiotic resistance in *Escherichia coli* isolated from the broiler houses and the pig nursery units.

**Additional file 3: Supplementary Table 2.** Antibiotic use at the broiler pilot farm.

## Data Availability

The datasets used and analysed during the current study are available from the corresponding author on reasonable request.

## Abbreviations

BKC: Benzalkoniumchloride
BPW: Buffered Peptone Water
C&D: Cleaning and disinfection
CFU: Colony forming units
DE broth: Dey Engley Neutralizing Broth
*E. coli*: *Escherichia coli*
ECOFF: Epidemiological cut-off
F: Formaldehyde
GA: Glutaraldehyde
H_2_O_2_: Hydrogen peroxide
ILVO: Flanders Research Institute for Agriculture, Fisheries and Food
MBC: Minimum Bactericidal Concentration
MHB: Mueller-Hinton broth
MIC: Minimum Inhibitory Concentration
PA: Peracetic acid
PCA: Plate Count Agar
QAC: Quaternary ammonium compound
TSB: Trypton Soya Broth
