# Supplementary material for: Repeated disinfectant use in broiler houses and pig nursery units does not affect disinfectant and antibiotic susceptibility in Escherichia coli field isolates
Source: BMC Vet Res. 2020 May 18;16:140. doi: 10.1186/s12917-020-02342-2 (PMC7236461; doi:10.1186/s12917-020-02342-2)
Supplement: Supplementary file 1 — Additional file 1: Supplementary Table 1. Statistical output of the logistic regression analysis. [file 12917_2020_2342_MOESM1_ESM.docx]

Supplementary Table 1: Statistical output of the logistic regression analysis showing no significant association between the used disinfectants and the antibiotic resistance of the isolates

|  | **Broiler house** | | | | | | **Pig nursery unit** | | | | | |
| --- | --- | --- | --- | --- | --- | --- | --- | --- | --- | --- | --- | --- |
|  |  | | | | | |  | | | | | |
| **Active disinfectant component →** | **A** | | **B** | | **C** | | **A** | | **B** | | **C** | |
|  | QAC-GA | | QAC-GA-F | | PA-H_2_O_2_ | | QAC-GA | | QAC-GA-F | | PA-H_2_O_2_ | |
| **Antimicrobial agent ↓** | Number of S/R isolates (%^*^) | P-value | Number of S/R isolates (%^*^) | P-value | Number of S/R isolates (%^*^) | P-value | Number of S/R isolates (%^*^) | P-value | Number of S/R isolates (%^*^) | P-value | Number of S/R isolates (%^*^) | P-value |
| Ampicillin | 4/12 (6%/19%) | 0.168 | 3/11 (5%/17%) | 0.992 | 11/23 (17%/36%) | 0.098 | 30/30 (21%/21%) | 0.264 | 19/14 (13%/10%) | 0.559 | 33/17 (23%/12%) | 0.245 |
| Sulfamethoxazole – Trimethoprim | 5/11 (8%/17%) | 0.241 | 6/8 (9%/13%) | 0.243 | 13/21 (20%/33%) | 0.093 | 41/19 (29%/13%) | 0.932 | 24/9 (17%/6%) | 0.708 | 35/15 (25%/10%) | 0.865 |
| Tetracycline | 13/3 (20%/5%) | 0.252 | 8/6 (13%/9%) | 0.097 | 24/10 (38%/16%) | 0.248 | 30/30 (21%/21%) | 0.621 | 20/13 (14%/9%) | 0.348 | 28/22 (20%/15%) | 0.544 |
| Ciprofloxacin | 11/5 (17%/8%) | 0.266 | 13/1 (20%/2%) | 0.129 | 28/6 (44%/9%) | 0.261 | - | | - | | - | |
| Nalidixic acid | 12/4 (19%/6%) | 0.368 | 13/1 (20%/2%) | 0.206 | 29/5 (45%/8%) | 0.272 | - | | - | | - | |
| Chloramphenicol | 16/0 (25%/0%) | 1.000 | 14/0 (22%/0%) | 1.000 | 31/3 (48%/5%) | 0.998 | 58/2 (41%/1%) | 0.777 | 33/0 (23%/0%) | 0.998 | 49/1 (34%/1%) | 0.477 |
| Azithromycin | - | | - | | - | | 60/0 (42%/0%) | 1.000 | 33/0 (23%/0%) | 1.000 | 49/1 (34%/1%) | 0.997 |
| Cefotaxime | - | | - | | - | | 60/0 (42%/0%) | 0.894 | 32/1 (22%/1%) | 0.997 | 49/1 (34%/1%) | 0.997 |
| Ceftazidime | - | | - | | - | | 60/0 (42%/0%) | 1.00 | 32/1 (22%/1%) | 0.997 | 50/0 (35%/0%) | 1.00 |
| Gentamicin | - | | - | | - | | 59/1 (41%/1%) | 0.216 | 30/3 (21%/2%) | 0.996 | 48/2 (34%/1%) | 0.997 |
|  |  |  |  |  |  |  |  |  |  |  |  |  |

QAC: quaternary ammonium compound; GA: glutaraldehyde, F: formaldehyde; PA: peracetic acid, H_2_O_2:_ hydrogen peroxide; -: no antibiotic resistance;

^*^ Percentage of the total number of poultry (n= 64) or pig isolates (n= 143)
