# Supplementary material for: Repeated disinfectant use in broiler houses and pig nursery units does not affect disinfectant and antibiotic susceptibility in Escherichia coli field isolates
Source: BMC Vet Res. 2020 May 18;16:140. doi: 10.1186/s12917-020-02342-2 (PMC7236461; doi:10.1186/s12917-020-02342-2)
Supplement: Supplementary file 2 — Additional file 2: Supplementary Fig. 1a and b. Prevalence of antibiotic resistance in Escherichia coli isolated from the broiler houses and the pig nursery units. [file 12917_2020_2342_MOESM2_ESM.docx]

C&D protocol

Supplementary Figure 1a: Prevalence of antibiotic resistance in *Escherichia coli* isolated from the broiler houses (expressed as percentage). Samples were taken after cleaning and disinfection (C&D) over a period of 6 production cycles: t0 (zero measurement, n= 3), t1 (after production cycle 1, n=18), t3 (after production cycle 3, n=40) and t5 (after production cycle 5, n=6). Monitoring of C&D took place from production cycle 1 onwards.

C&D protocol

**Supplementary Figure 1b: Prevalence of antibiotic resistance in *Escherichia coli* isolated from the pig nursery units (expressed as percentage). Samples were taken after cleaning and disinfection (C&D) over a period of 6 production cycles: t0 (zero measurement, n= 40), t1 (after production cycle 1, n=41), t3 (after production cycle 3, n=56) and t5 (after production cycle 5, n=46). Monitoring of C&D took place from production cycle 1 onwards**
