# Supplementary material for: Repeated disinfectant use in broiler houses and pig nursery units does not affect disinfectant and antibiotic susceptibility in Escherichia coli field isolates
Source: BMC Vet Res. 2020 May 18;16:140. doi: 10.1186/s12917-020-02342-2 (PMC7236461; doi:10.1186/s12917-020-02342-2)
Supplement: Supplementary file 3 — Additional file 3: Supplementary Table 2. Antibiotic use at the broiler pilot farm. [file 12917_2020_2342_MOESM3_ESM.docx]

Supplementary Table 2: Antibiotic use at the broiler pilot farm

| **Production cycle** | **Antibiotic administration** | **Product name** | **Active substance** | **Antibiotic class** |
| --- | --- | --- | --- | --- |
| 0 | Yes | Soludox 500 mg/g | Doxycycline | Tetracyclines |
| 1 | No |  |  |  |
| 2 | No |  |  |  |
| 3 | Yes | Emdotrim 10% SOL | Trimethoprim + Sulfadiazine | Trimethoprim & Sulphonamides |
|  |  | Solamocta 697 mg/g | Amoxicillin | Penicillines |
| 4 | No |  |  |  |
| 5 | No |  |  |  |
